# Evaluation of automatic discrimination between benign and malignant prostate tissue in the era of high precision digital pathology

**DOI:** 10.1186/s12859-022-05124-9

**Published:** 2023-01-03

**Authors:** Yauheniya Zhdanovich, Jörg Ackermann, Peter J. Wild, Jens Köllermann, Katrin Bankov, Claudia Döring, Nadine Flinner, Henning Reis, Mike Wenzel, Benedikt Höh, Philipp Mandel, Thomas J. Vogl, Patrick Harter, Katharina Filipski, Ina Koch, Simon Bernatz

**Affiliations:** 1 Institute of Pathology, Ludwig-Maximilians University Munich, Thalkirchner Str. 36, 80337 Munich, Germany; 2 Institute of Pathology, Charité - Universitätsmedizin Berlin, corporate member of Freie Universität Berlin, Humboldt-Universität zu Berlin, 10117 Berlin, Germany; 3 Molecular Bioinformatics Group, Institute of Computer Science, Faculty of Computer Science and Mathematics, Robert-Mayer-Straße 11-15, 60325 Frankfurt, Germany; 4 Dr. Senckenberg Institute for Pathology, Goethe University Frankfurt am Main, University Hospital Frankfurt, 60590 Frankfurt, Germany; 5 Wildlab, University Hospital Frankfurt MVZ GmbH, 60590 Frankfurt, Germany; 6 Frankfurt Institute for Advanced Studies (FIAS), 60438 Frankfurt, Germany; 7 Department of Urology, Goethe University Frankfurt am Main, University Hospital Frankfurt, 60590 Frankfurt, Germany; 8 Department of Diagnostic and Interventional Radiology, Goethe University Frankfurt am Main, University Hospital Frankfurt, 60590 Frankfurt, Germany; 9 Neurological Institute (Edinger Institute), University Hospital Frankfurt, 60590 Frankfurt, Germany; 10 German Cancer Consortium (DKTK), German Cancer Research Center (DKFZ), 69120 Heidelberg, Germany; 11 University Cancer Center (UCT) Frankfurt, Frankfurt, Germany; 12 Frankfurt Cancer Institute (FCI), University Hospital Frankfurt, 60590 Frankfurt, Germany

**Keywords:** Prostate cancer, Prediction, Quantitative features, Statistical analysis, Machine learning

## Abstract

**Background:**

Prostate cancer is a major health concern in aging men. Paralleling an aging society, prostate cancer prevalence increases emphasizing the need for efficient diagnostic algorithms.

**Methods:**

Retrospectively, 106 prostate tissue samples from 48 patients (mean age, $66\pm 6.6$ years) were included in the study. Patients suffered from prostate cancer (n = 38) or benign prostatic hyperplasia (n = 10) and were treated with radical prostatectomy or Holmium laser enucleation of the prostate, respectively. We constructed tissue microarrays (TMAs) comprising representative malignant (n = 38) and benign (n = 68) tissue cores. TMAs were processed to histological slides, stained, digitized and assessed for the applicability of machine learning strategies and open–source tools in diagnosis of prostate cancer. We applied the software QuPath to extract features for shape, stain intensity, and texture of TMA cores for three stainings, H&E, ERG, and PIN-4. Three machine learning algorithms, neural network (NN), support vector machines (SVM), and random forest (RF), were trained and cross-validated with 100 Monte Carlo random splits into 70% training set and 30% test set. We determined AUC values for single color channels, with and without optimization of hyperparameters by exhaustive grid search. We applied recursive feature elimination to feature sets of multiple color transforms.

**Results:**

Mean AUC was above 0.80. PIN-4 stainings yielded higher AUC than H&E and ERG. For PIN-4 with the color transform saturation, NN, RF, and SVM revealed AUC of $0.93\pm 0.04$, $0.91\pm 0.06$, and $0.92\pm 0.05$, respectively. Optimization of hyperparameters improved the AUC only slightly by 0.01. For H&E, feature selection resulted in no increase of AUC but to an increase of 0.02–0.06 for ERG and PIN-4.

**Conclusions:**

Automated pipelines may be able to discriminate with high accuracy between malignant and benign tissue. We found PIN-4 staining best suited for classification. Further bioinformatic analysis of larger data sets would be crucial to evaluate the reliability of automated classification methods for clinical practice and to evaluate potential discrimination of aggressiveness of cancer to pave the way to automatic precision medicine.

**Supplementary Information:**

The online version contains supplementary material available at 10.1186/s12859-022-05124-9.

## Introduction

Prostate cancer (PCa) is the second most common cancer and the fifth leading cause of cancer death in men [1]. Incidence rates vary across regions and PCa is the most frequently diagnosed cancer in men in 112 of 185 countries of the world [1]. One established risk factor is an advancing age [1]. Due to the demographic development of an aging society, we may expect an increasing PCa burden in the future [1]. Diagnosis of clinically significant prostate cancer is a challenging process. Most prostate cancers are slow-growing, a subset of prostate cancers has an aggressive clinical course and leads to death. Prostate cancer is usually suspected on the basis of screening procedures: digital rectal examination and/or prostate-specific antigen levels [2]. For definitive diagnosis, histopathological verification of PCa in prostate biopsy cores is required. Grading of PCa with the Gleason system is the strongest prognostic factor for clinical behavior and treatment response [2, 3]. Computerization and the efficient addressing of crucial cancer care touchpoints along the patient’s clinical pathway are major goals of current studies in the field of artificial intelligence (AI) in medicine [4]. Clinical decision support systems aim to assist physicians and other specialists in the analysis of patient’s data and diagnosis of diseases [5]. Quantitative imaging, machine learning (ML) algorithms, and AI have been proposed as potential solutions for assisting clinicians [6]. ML and AI have the potential to improve the accuracy and robustness of the diagnosis of PCa [7, 8]. Recent studies on PCa addressed the prediction of Gleason grade scores [9], the detection of PCa in biopsy specimen [10], the extraction of cancer stage from written reports in structured form [11], and the prediction of risk of PCa based on demographic characteristics [12]. Features extracted from digital images in pathology may have the potential to predict recurrence in PCa patients after surgery [13]. ML and AI have been used for cancer detection and grading based on whole image analysis in prostate biopsies [10, 14–18]. For the classification of benign and malignant tissues, multi-view boosting methods have been proposed [19]. The results have been compared to single-view classification and have reached a high area under the curve (AUC) score of 0.98 [19]. For a review of applications of deep learning to cancer detection, we refer to Pantanowitz et al. [20] and literature cited therein. Application of ML approaches may help in assisting physicians in the examination and prioritization of patient’s data, for a discussion, we refer to Bulten et al. [21].

The ground truth is usually based on visual inspection, evaluation and classification by expert pathologists. Besides hematoxilin and eosin (H&E) images, additional immunohistochemical workup can aid the urologic subspecialist in identifying and classifying cancer, e.g., see [8, 22]. Researchers in the field of digital pathology have put a considerable effort in the development and evaluation of methods especially for H&E images. Despite the well known advantages of immunohistochemical staining in daily standard of care clinical pathology, its possible additive predictive power is only scarcely studied and evaluated. Exploring the suitability of immunohistochemical stainings for automated classification tasks and machine learning models may provide an enhanced possibility for high precision digital pathology in the automatic classification of prostate cancer.

**Fig. 1:**
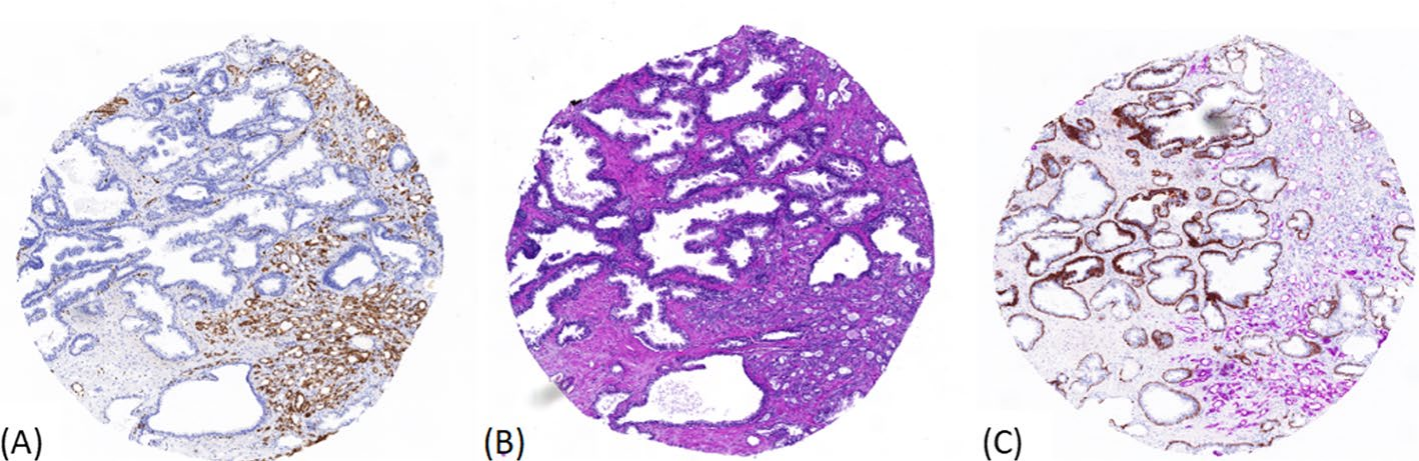
Exemplary cores with three stains: **A** ERG, **B** H&E, and **C** PIN-4. The diameter of a core is in round numbers 2 mm

In this work, we considered the staining methods, H&E, ERG, and PIN-4. H&E is a standard staining among others for cancer diagnosis [23]. ERG expression is a potential biomarker to predict the aggressiveness of prostate carcinoma with potential prognostic impact [24, 25]. PIN-4 is a cocktail of multiple markers and may help to distinguish between high-grade prostatic intraepithelial neoplasia and adenocarcinoma [26, 27]. Sabata et al. have investigated the identification and classification of glands in a whole slide image of PIN-4 stained prostate needle biopsy [27]. To our knowledge, no study has considered the AI–based prediction capability of PCa comparing the three commonly performed stainings: H&E, ERG, and PIN-4.

We retrospectively studied 106 tissue cores (malignant, n=38; benign, n=68) that we stained with three different methods, i.e., H&E, ERG, and PIN-4. The patient cohort included 48 patients (mean age, 66 ± 6.6 years), 38 patients with PCa, and ten patients with benign prostatic hyperplasia (BPH). Our purpose was to evaluate the suitability of basic image features based on the intensity distribution and the texture of the stained tissue cores to automatically differentiate between PCa tissue and benign tissue. We applied the open source software QuPath (version 0.2.0)  [28] for segmentation and feature extraction. We evaluated the predictive power of the features with three standard ML methods, i.e. neural network (NN), support vector machines (SVM) and random forest (RF) with and without optimization of hyperparameters and strategies for feature selection, e.g. recursive feature elimination (RFE) [29].

## Results

We compared malignant cores with benign cores. If not indicated otherwise, *p* values in the following were computed with the Wilcoxon–Mann–Whitney–U test [30] and corrected for multiple testing by the Benjamini–Hochberg procedure [31]. Exemplary, Fig. 1 shows cores with stain H&E, ERG, and PIN-4. Table 1 shows the number of features with false discovery rates (FDR): $p \le 0.05, p \le 0.01$, and $p \le 0.001$. For each core, QuPath computed five features of the intensity distribution and thirteen Haralick features based on the co-occurrence matrices for the texture [28], see Additional file 1: Table S5. Features of the intensity distribution and Haralick features were computed for each color channel. Color transforms were Red, Green, Blue, Saturation, Brightness, and Optical Density sum. For staining H&E and ERG, we also considered three stain specific color transforms, i.e., Hematoxilin, Eosin/Erg, and Residual, see Additional file 1: Sect. 1.4 *Digitalization*. Additionally, shape values, as, e.g., area, circularity, solidity, max/min diameter of the core, and the mean hue were determined per core. Exemplary, Fig. 1 shows cores with stain H&E, ERG, and PIN-4.

The values of the features are available as Additional files 2, 3, 4 and 5: Excel files. In Table 1 missing values and values with zero variance are excluded. None of shape features had FDR below $5\%$.

**Table 1:** Number of features for the H&E, ERG, and PIN-4 staining

Significance	H&E	ERG	PIN-4
All	166	166	117
$p \le 0.05$	114	127	76
$p \le 0.01$	105	111	63
$p \le 0.001$	92	94	44

We selected features with low FDR, $p \le 0.05, p \le 0.01$, and $p \le 0.001$

**Fig. 2:**
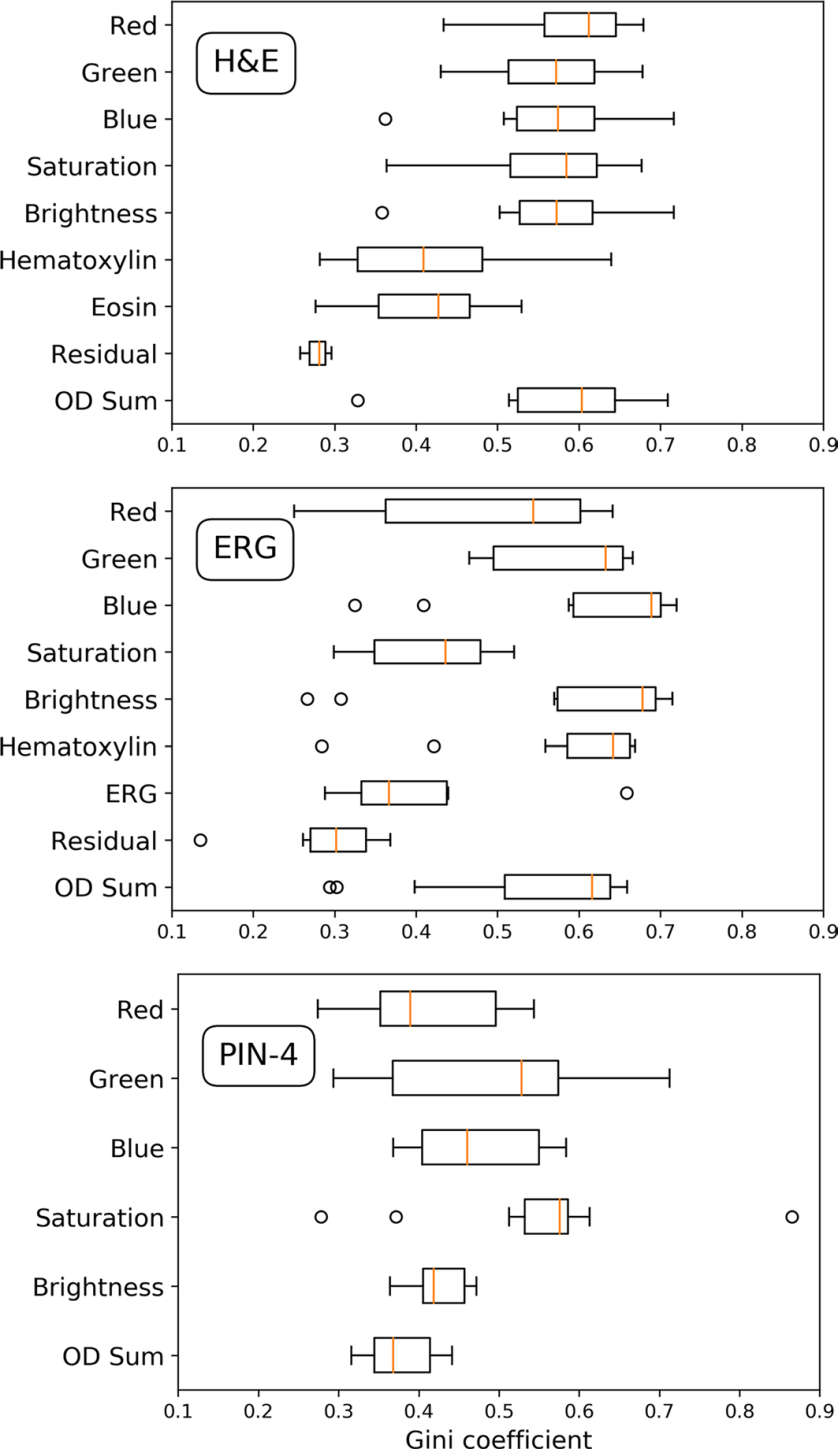
Predictive power of sets of features with $p \le 0.05$ for H&E staining (top), ERG staining (middle), and PIN-4 staining (bottom). Boxplots of Gini coefficients of features are given specifically for color transforms: Red, Green, Blue, Saturation, Brightness, and od sum. For H&E, additional box-plots of the stain–specific colors Hematoxilin, Eosin, and Residual are plotted. For ERG, additional box-plots of the stain–specific color transforms Hematoxilin, ERG, and Residual are plotted. The predictive power of features varies for the color transforms in each staining

We computed pairwise Pearson correlation coefficients of features and found features from different color transforms highly correlated. Due to highly correlated features from different color transforms, the selection of features with highest Gini score of the Wilcoxon–Mann–Whitney–U test was not a valuable strategy. To reduce the redundancy of features, we decided to consider, in the first step, only features of a single color transform for each stain. We compared the predictive power of features in the color transforms to select a representative color transform for each stain.

Figure 2 shows the boxplots of Gini coefficients of sets of features with $p \le 0.05$ specific for individual color transforms and stainings H&E (top), ERG (middle), and PIN-4 (bottom). A Gini coefficient of one corresponds to perfect predictive power whereas a Gini coefficient of zero corresponds to no predictive power, i.e., random choices. The predictive power of features varies for the stains. For H&E, the predictive powers of standard color transforms, Red, Green, Blue, Saturation, Brightness, and od sum, were preferable high with median Gini coefficients of in round numbers 0.6. For stain–specific colors Hematoxylin and Eosin, the median Gini coefficient drops to in round numbers 0.4. Residual has the lowest median Gini coefficient of in round numbers 0.3. For ERG, the median Gini coefficients are also preferable high. Differences in the color transforms are more pronounced than in H&E. Blue has a higher median Gini coefficient than Red and Green. Note that, counterstaining with hematoxylin produces a blue-purple signal for cell nuclei. High concentration of the protein ERG would manifest in a brownish nuclear signal, i.e., a high value of Red and Green. Surprisingly, the signal of counter-staining with hematoxylin has a higher median predictive power than the signal of ERG itself. Brightness has a higher predictive power than Saturation. Compared to ERG and H&E, the features of PIN-4 have rather low medians of Gini coefficient. An exceptional high Gini score of 0.865 gives, however, Maximum Saturation.

In the following, we denote features with $p \le 0.001$ as statistically significant. For H&E and ERG, we chose color transform Brightness and took 12 and 13 significant features, respectively. For PIN-4, we chose the 16 significant features of color transform Saturation, see Additional file 1: Sect. 1.5. On the stain–specific sets of features, we applied three ML algorithms: support vector machines classifier (SVM), neural networks (NN), and random forest (RF). We transformed the categorical labels malignant and benign to dichotomous labels 1 (positive) and 0 (negative), respectively. Monte Carlo cross-validation with 100 random splits into 70% training set and 30% test determined the mean AUC of the receiver operating characteristic (ROC) curve. We applied stratification at patient (n = 48) level to avoid having cores coming from the same patient present in both training and validation.

**Table 2:** Mean AUC for three ML algorithms, support vector machines classifier (SVM), neural network (NN), and random forest (RF), trained on the sets of features from three stains, H&E (n=12), ERG (n=13), and PIN-4 (n=16), see text

	SVM		RF		NN	
	Default	Tuned	Default	Tuned	Default	Tuned
H&E	$0.80 \pm 0.07$	$0.90 \pm 0.06$	$0.82 \pm 0.08$	$0.82 \pm 0.08$	$0.84 \pm 0.07$	$0.89 \pm 0.07$
ERG	$0.83 \pm 0.06$	$0.84 \pm 0.07$	$0.85 \pm 0.06$	$0.85 \pm 0.05$	$0.85 \pm 0.06$	$0.86 \pm 0.05$
PIN-4	$0.92 \pm 0.05$	$0.93 \pm 0.06$	$0.91 \pm 0.06$	$0.92 \pm 0.05$	$0.93 \pm 0.04$	$\mathbf {0.94} \pm 0.04$

**Fig. 3:**
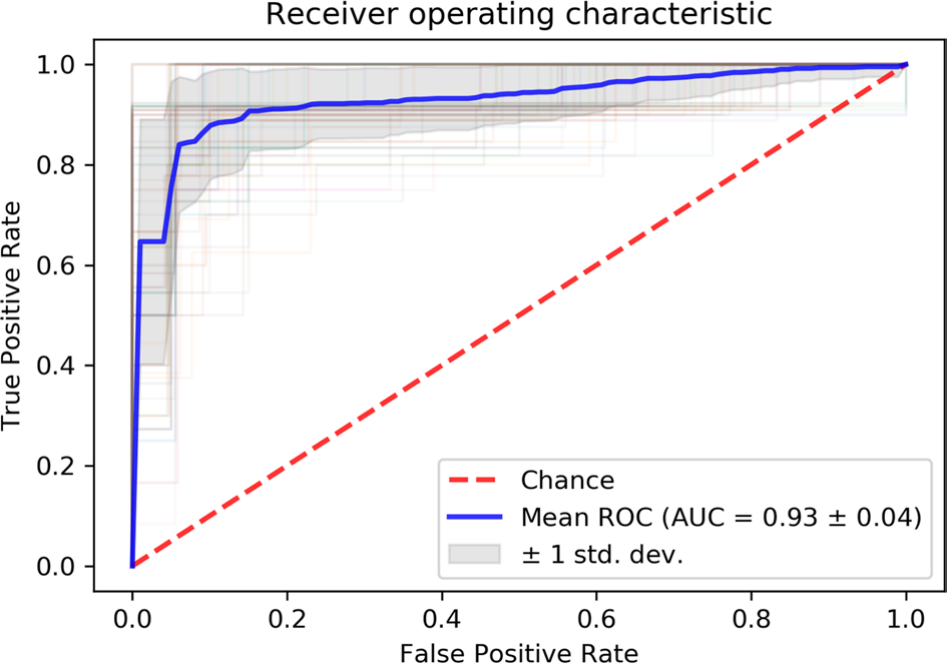
ROC curves of Monte Carlo cross-validation with 100 random splits. The solid (blue) curve denotes the mean ROC curve and the shaded gray area highlights its standard deviation. NN uses 16 significant features of the color transform Saturation of stain PIN-4 to compute the curves, see text. The AUC $0.93 \pm 0.04$ of the mean ROC curve is preferable high and demonstrates the predictive power of the features of the color transform Saturation of stain PIN-4

Table 2 shows, in the rows denoted by ”default”, the mean AUC with standard deviation. The mean values of AUC are preferable high, $0.80 \le \text {AUC}\le 0.93$, and demonstrate the predictive power of the three groups of features for H&E, ERG, and PIN-4, respectively. The group of features of PIN-4 yields the best results. NN performs with the mean AUC of $0.93\pm 0.04$ for the group of selected features of PIN-4. The range of values of AUC, however, does not exceed the values that might be expected from the Gini score of individual features. Note that, the Gini score of 0.865 of a single feature, Maximum Saturation of PIN-4, corresponds to an AUC of 0.93. Exemplary, Fig. 3 shows the mean ROC curve of a Monte Carlo 100 random–split cross-validation for NN and the 16 significant features in color transform Saturation of stain PIN-4.

To enhance the performance of the algorithms, we applied an exhaustive grid search to optimize their hyperparameters, see Additional file 1: Sect. 1.8. Table 2 gives, in the rows denoted by ”tuned”, the mean AUC yielded by the classifiers with optimized parameters. The optimization of parameters improves the results for SVM and features of H &E, i.e., the mean AUC increases from 0.80 to 0.90. For all other combinations of ML algorithms and feature sets, the optimization yields none or only minor improvement less than $\Delta \text {AUC} \le 0.05$.

Since parameter optimizations yielded only small improvements of predictive power, we tested whether other groups of features may yield better results. We applied recursive feature elimination (RFE) [29] to select sets of non-redundant features from all color transforms of a stain. To reduce the computational expense of RFE, we removed shape features and redundant features by setting thresholds for the Pearson correlation coefficient. We chose thresholds for the Pearson correlation as large as possible but with the restriction to make the computation feasible on a conventional laptop computer with i7 processor and 8 GByte memory. The number of non-redundant features were $n=84$ (PIN-4, Pearson correlation $\le 0.95$), $n=104$ (H&E, Pearson correlation $\le 0.99$), and $n=53$ (ERG, Pearson correlation $\le 0.95$). We applied RFE with successively increased numbers of top features and saved the set with highest accuracy. If two sets yielded identical accuracy, we favoured the smaller set. RFE yielded 25 features (mean accuracy $0.778\pm 0.070$), 9 features (mean accuracy $0.844\pm 0.064$), and 5 features (mean accuracy $0.979\pm 0.032$) for H&E, ERG, and PIN-4, respectively. To compute accuracy values for the three sets of selected features, we applied NN and Monte Carlo cross-validation with stratification at patient (n=48) level and 100 random splits into 70% training set and 30% test. With only five selected features, PIN-4 reached the best mean accuracy of 97.9%. Additional file 1: Table S2 lists AUC values for SVM, RF, and NN. Compared to the corresponding AUC scores in Table 2, either no or minor improvement can be observed for stains H&E and ERG. For stain PIN-4, NN computed nearly perfect AUC of $0.992\pm 0.012$, see Additional file 1: Fig. S1 for the mean ROC curve.

## Discussion and conclusion

Automatically extracted features of the texture and intensity distribution of stained TMA turned out to be highly valuable to distinguish between malignant and benign tissue of the prostate gland. High predictive power could be shown already for individual features, as, e.g., Maximal Saturation of the PIN-4 stain. The three staining protocols H&E, ERG, and PIN-4 yielded different predictive power.

Cost-effective and simple H&E revealed promising results. Our results for H&E, e.g., AUC$=0.90\pm 0.06$, SVM, tuned, were not impressive when compared to results of previous studies applying deep learning. Recently, AUC of in round numbers 0.99 have been reported for the application of convolutional neural networks to H&E stained TMA [32] and whole slide images [20]. Application of deep learning requires large number of data. Its black-box characteristic may be seen as possible drawback for medical decision making [32–34].

ERG stain revealed results of similar quality as H&E. Averaged over all cores, staining high expression of the proto-oncogene ERG seems to give no advantage compared to a simple H&E stain. Elevated expression of ERG occurs only in a subset of approximately 50% of PCa cases [35, 36]. The high fraction of PCa cases with no elevated expression of ERG may significantly reduce the sensitivity and accuracy of ERG as a PCa-identifying biomarker in a machine learning approach.

In individual cases, the ERG stain may give valuable additional information.

We abstained from testing the possible advantage of the combination of features of ERG stain with features of other stains, e.g., H&E and PIN-4.

In our study, PIN-4 showed the most accurate results. For PIN-4, NN yielded AUC$= 0.94\pm 0.04$ for features extracted from color transform Saturation. PIN-4 has been reported to be useful in distinguishing prostatic adenocarcinoma from the benign mimickers [27, 37, 38]. We applied the stain PIN-4 as a cocktail of two antibodies, a brownish signal for high molecular weight cytokeratins, and a second, reddish signal for the protein alpha-methylacyl-CoA racemase (AMCAR,P504S). P504S is a biomarker for prostate adenocarcinoma [39, 40]. Positive staining with a monoclonal antibody to high molecular weight cytokeratins has been shown to be of value in distinguishing between well-differentiated, small-acinar prostatic adenocarcinoma and its mimics [41]. Therefore, the superior performance of PIN-4 compared to H&E and ERG is not surprising. To our knowledge, PIN-4’s potential application for automatic stratification of PCa in medical AI has not been tested up to now. In AI applications, staining with PIN-4 has been merely used as a preferable additional immunohistochemical workup to generate the ground truth by visual inspection [8, 22].

In the year 2010, Sabata et al. [27] studied the potential of computer–aided diagnoses of PIN-4 stained needle biopsies. Their algorithm has identified the glands in the tissue and has classified the glands by the three simple criteria:

1. “If gland has only the brown basal staining then the tumor is benign”,
2. “If gland has both the red racemace and the brown basal staining then it is classified as high-grade prostatic intraepithelial neoplasia (HGPIN)”,
3. “If gland has only the red racemace then it is classified as adenocarcinoma”.

Sabata et al. have discussed several possible sources of miss-classification.

For small glands, a big variation in the intensity of racemace staining may cause recognition of the red staining to be error–prone. It has been important to not merge a gland with the surrounding glands or the diagnosis would have been incorrect. Note that, automated object segmentation is a task and a potential source of missclassification. In view of recent studies, the three simple rules proposed by Sabata et al. are not likely to be able to account for possible heterogeneity of staining of benign and malignant tissue. It is possible that benign glands may show some weak to moderate AMCAR expression and, on the other hand, it is not a necessity for prostate cancer to be AMCAR–positive (especially high grade subtypes can be negative, and inter- and intratumoral heterogeneity can occur) [42, 43]. For benign tissue, e.g., tissue of atypical adenomatous hyperplasia (AHH), high expression of AMCAR has been reported in up to more than 50% of the cases [44].

In our approach, we used intensity and texture features of a core with, in general, multiple glands for its classification. Ranking features by their predictive power, i.e., their Gini coefficient, we found Maximum Saturation of PIN-4 by far the top feature. High Maximum Saturation indicated malignancy: 87% , i.e., 33 out of 38, malignant cores compared to only 6%, i.e., four out of 66, benign cores had Maximum Saturation above 0.953.

Brownish membranous signal for cytokeratin yielded low values of Saturation whereas a pure reddish cytoplasmic signal for AMACR yielded high values of Saturation. Maximum Saturation identified a local region (2 µm resolution) with reddish cytoplasmic signal and no brownish membranous signal for cytokeratin. The presence of such a local region inside a core with high and pure reddish signal was a good condition to diagnose malignancy, see, e.g., Additional file 1: Fig. S6. Our finding of 87% malignant cores and 6% benign cores with Maximum Saturation above 0.953 is in line with Murphy et al. [42] who described 91% and 11% AMCR positivity of prostate cancers and benign tissues, respectively.

Within our data set, we found five malignant cores with low values of Maximum Saturation. Additional file 1: Fig. S2 shows the five malignant cores with Maximum Saturation values below 0.953. Based on the threshold value 0.953 for Maximum Saturation, these five cores would had been missclassified as benign cores. The cores have low reddish signal if compared to cores with high Saturation as shown, e.g., in Additional file 1: Fig. S6. The low reddish signal for some malignant cores might be a results of the known potential inter- and intra-tumoral heterogeneity of prostate cancer AMACR positivity [42]. Heterogenous AMCAR expression is significantly associated with increased Gleason score and poorly differentiated tumors [42]. Respectively, the two tumor cores in Additional file 1: Fig. S2 A, B show AMCAR negative high grade prostate cancers. The tumor cores in Additional file 1: Fig. S2 C–E show low grade prostate cancers with low AMCAR expression.

In our data set, we found also four benign cores with values of Maximum Saturation above 0.953, see Additional file 1: Fig. S3. Staining artefacts, see, e.g., core (A), or intense dark brown staining, see, e.g., cores (B–D), lead to high values of Maximum Saturation. Three of these four benign cores were on the same slide, RPX3. Averaged exclusively over benign cores, Maximum Saturation $0.910\pm 0.043$ of slide RPX3 was higher than Maximum Saturation $0.839\pm 0.029$ and $0.873 \pm 0.039$ of slides RPX1 and RPX2, respectively. Compared to the mean value of Maximum Saturation $0.967\pm 0.037$ of malignant cores, the mean values of benign cores on each slide were low. Slide–to–slide variations and the potentially heterogenous AMCAR expression of malignant and benign glands, however, made it problematic to determine a global threshold for a classification based solely on Maximum Saturation. Inclusion of definite benign tissue with and without AMCAR expression on a slide as reference for benign saturation values might be a potential solution.

In a previous study [45], the biological variance between punches within the same tissue, i.e., intra-tissue variance, has been shown to be of similar magnitude as the variation between tissues and patients. In accordance with this finding, we found no difference between an approach with and without stratification at patient level. Numerical results were, beside insignificant statistical variations, identical with and without patient stratification. For our data set, the assumption of independence of cores from one patient produced no bias in the numerical results. For other data sets, the assumption of independence of cores from one patient may not be a valid strategy and hence, we presented only numerical values with stratification at patient level to avoid having cores coming from the same patient present in both training and validation sets.

Spots located on the same slide may share bias. Exemplary, we chose the cores of two slides as train set and the cores of the third slide as test set. We observed no significant drop in model performance, e.g., SVM trained on five features of PIN-4 obtained preferable values of $100\%$ and 97.5–$100\%$ for sensitivity and AUC, respectively. Future studies with a larger number of slides may give a more conclusive picture of the relevance of stratification at the level of slides.

Our patient cohort was biased towards older population (mean age, $66\pm 6.6$ years). Since age-associated changes in AMACR expression has been reported in nonneoplastic prostatic tissues [46], age may be a valuable additional feature for populations with heterogeneous age distributions.

For PIN-4, the algorithms SVM and NN yielded even higher AUC of $0.997\pm 0.009$ with five features that were extracted not only from Saturation but also from two additional color channels, Red, and Blue. The relevance of Red and Blue probably arose from the role of reddish signal for the protein alpha-methylacyl-CoA racemase and the role of brownish signal for high molecular weight cytokerine, respectively. Saturation contributed to high value of AUC but, interestingly, not Maximum Saturation but Haralick Sum Variance of Saturation was one of the five selected features.

In contrast to the local property Maximum Saturation, the Haralick Sum Variance measured a global property, i.e., a normalized value averaged over all neighbored pixel pairs of a core. In view of the slide–to–slide variation of staining in our data set, the application of a global and normalized Haralick feature may be more robust than the local measure of Maximum Saturation.

Notice that, our feature selection procedure may suffer from overfitting, for a discussion of a possible bias we refer to Demirciouglu [47]. The application of strategies to avoid overfitting in feature selection requires a larger data set. Our AUC values of classification without feature selection may be more reliable and relevant for applications to independent data sets. Despite the possible bias by overfitting, the sets of selected features on its own may be valuable for studies with an independent data set.

Analysis of performance variation, based on different combinations of features from different stainings was beyond the scope of this manuscript, due to sample size limitations. A valid analysis of performance improvement based on multiple stainings, i.e. ERG and PIN-4, would require a larger data set. Of note, we revealed PIN-4 as best suited staining for discriminating between benign and malignant prostate tissue and therefore its inclusion in digitized pathology may be recommendable. Dedicated analysis of the relevance of individual texture features for given stainings as, e.g., ERG and PIN-4, may provide valuable insights into the phenotype of PCa.

From what we have pointed out, we draw the conclusion that the automatic extraction of imaging features from stained prostate tissue slides may be feasible to discriminate malignant and benign tissue with high accuracy. PIN-4 staining was best suited for the classification. In future studies, it may be worthwhile to test deep-learning algorithms on PIN-4 images, to evaluate images of different image resolutions, to develop a suitable color model for PIN-4, and to study strategies to correct for slide–to–slide differences in staining, e.g., by a reference tissue cores or automated slide specific normalization techniques.

## Material and methods

All methods were carried out in accordance with relevant guidelines.

The patient cohort comprised 48 patients (mean age, $66~\pm ~6.6$ years), 38 patients with PCa and ten patients with BPH, for details screening process and inclusion criteria, we refer to Bernatz et al. [48] and Additional file 1: Sect. 1.1. In total, 106 cores of prostate tissue (malignant, n=38; benign opposite site of PCa-patients, n=38; repetitive punches of HoLEP tissue, n=30) were punched to construct three TMA. TMA 1, TMA 2, and TMA 3 contained 42, 42, and 22 cores of prostate tissue, respectively, see Additional file 1: Fig. S4. Afterwards, we stained the TMA with H&E, ERG, and PIN-4, respectively, see Fig. 1.

We digitized the histology slides with a digital slide scanner (Sysmex GmbH, Germany, resolution 2 µm per pixel). The image processing included de-arraying of the TMA and computation of feature values for each core. Out of a total number of 318 stained cores, 106 cores times three stains, three cores had to be excluded from our analysis due to poor staining quality. The three excluded cores could not be recognized and processed by QuPath. Additional file 1: Table S4 gives the final number of processed malignant and benign cores. We refer to Additional file 1: Sects. 1.2–1.4, for details of preparations of TMA, their histological staining, digitalization, and the computation of features.

### Software

We processed tissue microarrays with the open–source software for digital pathology and whole slide image analysis QuPath (version 0.2.0) [28]. We wrote Python scripts (Python version 3.7.6) [49]) in Jupyter Notebook [50]. We used modules from the scipy package (version 1.4.1) [51] for statistical calculations and applied ML algorithms from the scikit-learn library (version 0.22.1) [52]. For recursive feature elimination, we applied the implementation RFECV [29] from the scikit-learn library (version 0.22.1).

## Supplementary Information

**Additional file 1:** Supplementary data.

**Additional file 2:** Classification labels (benign=0; malignant=1) for each tissue core.

**Additional file 3:** Feature values for ERG stain.

**Additional file 4:** Feature values for H&E stain.

**Additional file 5:** Feature values for PIN-4 stain.

## Data Availability

All data generated or analysed during this study are included in this published article and its Additional files.
