# Supplementary material for: Evaluation of automatic discrimination between benign and malignant prostate tissue in the era of high precision digital pathology
Source: BMC Bioinformatics. 2023 Jan 3;24:1. doi: 10.1186/s12859-022-05124-9 (PMC9809030; doi:10.1186/s12859-022-05124-9)
Supplement: Supplementary file 1 — Additional file 1: Supplementary data. [file 12859_2022_5124_MOESM1_ESM.pdf]

SUPPLEMENTAL MATERIAL TO

Evaluation of automatic discrimination between benign and malignant prostate tissue in the era of high precision digital pathology

Yauheniya Zhdanovich[1,2], Jörg Ackermann[3], Peter J. Wild[4,5,6], Jens Köllermann[4], Katrin Bankov[4], Claudia Döring[4], Nadine Flinner[4], Henning Reis[4], Mike Wenzel[7], Benedikt Höh[7], Philipp Mandel[7], Thomas J. Vogl[8], Patrick Harter[9], Katharina Filipski[9,10,11,12], Ina Koch[3] and Simon Bernatz[4,8,12*]

*Correspondence:
Simon.Bernatz@kgu.de
[8]Department of Diagnostic and Interventional Radiology, Goethe University Frankfurt am Main, University Hospital Frankfurt, 60590 Frankfurt am Main, Germany
Full list of author information is available at the end of the article

List of Abbreviations

AUC area under the curve.

BPH benign prostatic hyperplasia.

ERG stain with a antibody for ERG protein (ERG for Erythroblast transformation-specific Related Gene).

FDR false discovery rate.

HoLEP Holmium Laser Enucleation of the Prostate.

H&E hematoxilin and eosin.

ISUP international society of urological pathology score.

ML machine learning.

NN neural networks.

PCa Prostate Cancer.

PIN-4 double stain with two antibodies, AMACR(P504S), and high molecular weight cytokeratin.

QuPath open source software for digital pathology and whole slide image analysis QuPath (version 0.2.0) [1].

RF random forest.

RFE recursive feature elimination.

ROC receiver operating characteristic.

RPX radical prostatectomy.

SVM support vector machines.

TMA tissue microarray.

UCT University Cancer Center Frankfurt.

1.1 Patient Cohort

Tissue/tumor samples and patient data were provided by the University Cancer Center Frankfurt (UCT). Written informed consent was obtained from all patients and the study was approved by the institutional Review Boards of the UCT and the Ethical Committee at the University Hospital Frankfurt (project-number: SUG-4-2018). The project expands on the results of Bernatz *et al.* [2] and in total 418 patients with confirmed PCa who were treated with radical prostatectomy (RPX) between 2014 and 2019 were screened for study inclusion [2]. In the current study, contrary to Bernatz *et al.* [2] patients with neoadjuvant therapy prior to RPX (n=6) were included and 1 PCa patient had to be excluded due to an insufficient amount of PCa-tissue leading to final study cohort of 38 PCa patients, see Bernatz *et al.* [2] for details. As negative control, 10 patients with benign prostatic hyperplasia (BPH) who were treated with Holmium laser enucleation of the prostate (HoLEP) were used. Inclusion criteria for the HoLEP cohort was (I) suffering from BPH and having received (II) treatment with HoLEP without (III) cancerous tissue in the HoLEP tissue. The final patient cohort comprised of 48 patients (mean age, 66 $\pm$ 6.6 years), 38 patients with PCa and ten patients with BPH.

1.2 Preparation of tissue microarrays

Prior to the TMA establishment, all whole slide specimen were annotated by an uropathologist (JK, 10 years of experience) to (I) delineate the areas of PCa index lesion with highest international society of urological pathology (ISUP) score, (II)

benign tissue at the opposite site of the respective PCa slides, and (III) benign HoLEP specimen. In total, 48 paraffin-embedded tissue samples from our patient cohort were used to construct the TMAs by punching 106 representative tissue cores from the paraffin blocks. The representative punch-locations were annotated on respective H&E-slides of each tissue block which was used as a mask to identify respective regions on the tissue block. We punched a core (2 mm diameter) from the index lesion of each PCa-tissue ($n = 38$). As matched-controls we used a tissue punch from the benign opposite site of each PCa whole gland specimen ($n = 38$) and three independent tissue-punches from each patient who was treated with HoLEP for benign prostatic hyperplasia ($n = 10 \times 3 = 30$). In total, 106 cores of prostate tissue (malignant, n=38; benign opposite site of PCa-patients, n=38; repetitive punches of HoLEP tissue, n=30) were punched to constuct TMA 1-3. TMA 1, TMA 2, and TMA 3 contained 42, 42, and 22 cores of prostate tissue, respectively, see, e.g., Figure S4. TMA blocks were cut into 3 μm thick slices and placed on an adhesive glass slide. Unstained slides were stained with H&E as well as with immunohistochemical staining ERG and PIN-4.

1.3 Histological staining

For immunohistochemistry (IHC), we used DAKO FLEX-Envision Kit (Agilent, Santa Clara, CA, US) and the fully automated DAKO Omnis staining system (Agilent, Santa Clara, CA, US) according to manufacturer´s instruction. We applied heat induced epitope retrieval at 97°C in high pH buffer, EnV FLEX TRS High pH Buffer (Agilent, Santa Clara, CA, US). Afterwards we applied immunohistochemical epitope staining for 20 min by either PIN-4 double stain or ERG single stain. PIN-4 co-stained high molecular weight cytokeratin, DAKO primary antibody Cytokeratin High Molecular Weight (Clone 34betaE12, GA051, ready to use dilution, Agilent, Santa Clara, CA, US), and protein alpha-methylacyl-CoA racemase, AMACR (Clone 13H4, GA060; ready to use dilution, Agilent, Santa Clara, CA, US). ERG contained single-staining ERG primary antibody (GA659, Clone EP111, ready to use dilution, Agilent, Santa Clara, CA, US). For epitope visualization, we applied DAKO EnVision™ FLEX DAB+ and Magenta Substrate Chromogen System (Agilent, Santa Clara, CA, US). PIN-4 double stain produced a brownish membranous signal for cytokeratin and reddish cytoplasmic signal for AMACR [3, 4]. ERG single stain produced a brownish nuclear signal for high concentration of the protein ERG. After immunohistochemical staining, we used hematoxylin, DAKO hematoxylin solution (Agilent, Santa Clara, CA, US), for counterstaining. Hematoxylin produced blue-purple signal for cell nuclei.

For hematoxylin and eosin stain (H&E), slides were automatically processed using Tissue-Tek Prisma Plus staining device (Sakura Finetek) and Mayer´s Hematoxylin (AppliChem, Darmstadt, Germany) and Eosin (Waldeck, Münster, Germany) according to manufacturer´s instruction. H&E produced blue-purple signal for acidic cell nuclei and a pink signal for alkaline cytosolic and extracellular structures. Figure S5 shows three exemplary cores that are stained with H&E, ERG, and PIN-4, respectively.

Chromosomal rearrangements, or mutations, involving the ERG protein are highly specific for prostate cancer, occurs in 40–50% cases of prostate cancer and lead to

the elevated expression of the ERG gene [5, 6]. In the presence of ERG expression, ERG antibodies yield a positive staining of cancer nuclei and a faint cytoplasmic staining [7].

1.4 Digitalization

We digitised the histologic slides with a digital slide scanner (Sysmex GmbH, Germany, resolution 2 μm per pixel). We processed the images with an open source software for digital pathology and whole slide image analysis, QuPath (version 0.2.0) [1]. The image processing included de-arraying of the TMA and computation of feature values for each core.

Out of a total number of 318 stained cores, 106 cores times three stains, three cores had to be excluded from our analysis due to poor staining quality. The three excluded cores could not be recognized and processed by QuPath. For the detailed number of processed malignant and benign cores, we refer to Table S4.

QuPath extracted a grey-scale image for each color transform Red, Green, Blue (RGB color model), Hue, Saturation, Brightness (HSB color model) and Optical Density sum (OD–sum), see [8, 1] for a detailed description of the color models. We applied color deconvolution of QuPath to correct for minor variations between individual slides. QuPath implements the color deconvolution method of Ruifrok and Johnston (2001) [8]. The color deconvolution separates three stains based on the background values and a stain vector for each stain. QuPath provides default stain vectors, e.g., to characterize hematoxylin, eosin or 3,3'-diaminobenzidine (DAB). For each slide, we manually selected a representative region containing relatively clear examples of the stains and background. QuPath adjusted the stain vectors based on the rgb values of the pixels in the representative region. For H&E, color deconvolution determined slide specific color vectors for Hematoxylin, Eosin, and Residual. For ERG, color deconvolution determined slide specific color vectors for Hematoxylin, ERG, and Residual. For PIN-4, color deconvolution determined slide specific color vectors that varied strongly from slide to slide and an unique assignment to stains was not possible. The limited ability of automated deconvolution to account for more than two stains may be the reason for the failure of color deconvolution in the case of the triple staining PIN-4.

We chose the set of standard features of QuPath. QuPath computed standard features for each core, as, e.g., five features of the intensity distribution, thirteen Haralick features based on the co-occurrence matrices for the texture, and shape values, as, e.g., area, circularity, solidity, max/min diameter of the core [1]. Within QuPath, the Haralick features are denoted by abbreviations F0–F12, for a list of abbreviations of features we refer to Table S5. Features of the intensity distribution and Haralick features were computed for color transforms Red, Green, Blue, Saturation, Brightness, and OD–sum. For color transform Hue, only the mean intensity was computed. For staining H&E and ERG, features of the intensity distribution and Haralick features were computed also for the three stain specific color transforms Hematoxylin, Eosin/ERG, and Residual. In total the maximum number of features, n_{features}, per core was

$$n_{\text{features}} = 18 \times n_{\text{color}} + n_{\text{shape}} + 1 \ .$$

For staining PIN-4, the number of color transforms was $n_{\text{color}} = 6$. For staining H&E and ERG, the number of color transforms was $n_{\text{color}} = 9$. The number of shape features was $n_{\text{shape}} = 8$ and one feature was the mean hue. None of shape features had FDR below 5% , i.e., was statistically significant. After elimination of features with missing values or zero variance, we recorded 166, 166, and 117 features values for a core stained with H&E, ERG, and PIN-4, respectively. The values of the features are available as supplemental Excel files.

1.5 Stain specific features sets

We denoted features with $p \leq 0.001$ (Wilcoxon-Mann-Whitney U test) as statistically significant. For H&E and ERG, we chose color transform Brightness and took 12 and 13 significant features, respectively. For PIN-4, we chose the 16 significant features of color transform Saturation. The significant features were, ordered by decreasing Gini score:

- H&E staining, color transform Brightness, 12 significant features: F11, F12, F0, F4, F2, F9, F8, Median, Mean, F5, F7, F10.
- ERG staining, color transform Brightness, 13 significant features: Mean, F5, F7, F8, Median, F10, F4, F0, F9, F1, Std.dev., F3, F6
- PIN-4 staining, color transform Saturation, 16 significant features: Max, F12, F0, F7, F8, Median, Mean, F5, F9, F2, F4, F6, Std.dev., F3, F10, F1.

For the full names of the thirteen Haralick features F0–F12, we refer to Table S5.

1.6 Features sets of recursive feature elimination

We applied the recursive feature elimination method of Guyon *et al.* (2002) [9], implemented as function RFECV in the scikit-learn library (version 0.22.1). We successively increased numbers of top features and saved the set with highest accuracy. If two sets yielded identical accuracy, we favoured the smaller set. For the three stains, RFE yielded the sets:

- H&E staining, 25 features with mean accuracy 0.780 ± 0.061:
  Hematoxylin: F1, F8, Eosin: F1, F3, F6, Residual: Max, F2, F6, F11, F12, Green: F1, F5, F7, F10, Blue: F1, F7, F8, F10, Brightness: Min, Saturation: F1, F8, F10, and OD Sum: Max, F8, F10.
- ERG staining, 9 features with mean accuracy 0.829 ± 0.066:
  Red: Median, F3, Green: Mean, F1, Brightness: F1, F6, F3, and OD Sum: F8, F7
- PIN-4 staining, 5 features with mean accuracy 0.973 ± 0.037:
  Red: Median, F6 (Haralick Sum variance), Blue: F3 (Haralick Sum of squares), F1 (Haralick Contrast), and Saturation: F6 (Haralick Sum variance).

For the full names of the thirteen Haralick features F0–F12, we refer to Table S5. To compute reference accuracy values for the three sets of selected features, we applied NN, stratification at patient level, and Monte Carlo cross-validation with 100 random splits into 70% training set and 30% test.

1.7 Statistical Analysis

We applied the non-parametric Mann–Whitney U test [10] to compare two unpaired groups, e.g., malignant versus benign tissue. The Mann-Whitney U test computes

the U statistic of two samples $x_1, ..., x_m$ and $y_1, ..., y_n$ as

$$U = \sum_{i=1}^{m} \sum_{j=1}^{n} (I[y_j < x_i] + \frac{1}{2} I[y_j = x_i]),$$

where $I[A] = 1$ if the event A is true, and $I[A] = 0$ otherwise [11]. The U statistic determines the significance of the inequality of the two groups and the Gini coefficient

$$\text{Gini} = \left| \frac{2\,U}{m\,n} - 1 \right|$$

with m, n the sizes of the two groups [12, 13]. The Gini coefficient can be scaled to the area under the receiver operating characteristic (ROC) curve (AUC) [12]

$$\text{AUC} = \frac{\text{Gini} + 1}{2} \ .$$

The AUC represents the probability that a randomly chosen subject is correctly classified. An AUC of 0.5 (Gini = 0) corresponds to a random choice and an AUC of 1.0 (Gini = 1) corresponds to a perfect discrimination between the two groups [14, 12]. To correct the significance for multiple testing, we applied a Bonferroni adjustment and computed the false discovery rate (FDR) by the Benjamini-Hochberg procedure [15].

1.8 Parameter optimization

We optimized hyperparameters with the function `model_selection.GridSearchCV` of the scikit-learn library (version 0.22.1) [16] in Python. For SVM, we adjusted the regularization parameter, C, kernel coefficient, γ, and kernel. For RF, we adjusted number of trees in the forest, `n_estimators`, the maximum depth of the tree, `max_depth`, randomness of the bootstrapping of the samples, `random_state`, the minimum number of samples required to split an internal node, `min_samples_split`, and the minimum number of samples required at a leaf node, `min_samples_leaf`. For NN, we adjusted structure of a network, `hidden_layer_sizes`, activation function, `activation`, learning rate schedule for weight updates, `learning_rate`, solver for weight optimization, `solver`, regularization term, α, maximum number of iterations, `max_iter`, and random number generation for weights and bias initialization, `random_state`. For the customized hyperparameters, we refer to Table S1.

1.9 Software

We processed tissue microarrays with the open source software for digital pathology and whole slide image analysis QuPath (version 0.2.0) [1]. We wrote Python scripts (Python version 3.7.6) [17]) in Jupyter Notebook [18]. We used modules from the scipy package (version 1.4.1) [19] for statistical calculations and applied ML algorithms from the scikit-learn library (version 0.22.1) [16].

Tables

Table S1: Optimized hyperparameters of the classifiers support vector machines classifier (SVM), neural networks (NN), and random forest (RF). We performed an exhaustive grid search to enhance the precision of the classifiers for each individual staining, H&E, ERG, and PIN-4.

	SVM	RF	NN
H&E	$C = 1000$,<br>$\gamma = 0.00001$,<br>kernel='linear',<br>probability=True	random_state= 1,<br>max_depth= 15,<br>n_estimators= 500,<br>min_samples_split= 2,<br>min_samples_leaf= 1	hidden_layer_sizes= $(1, 100)$,<br>learning_rate='constant',<br>random_state= 1,<br>solver='lbfgs'
ERG	$C = 0.1$,<br>$\gamma = 0.005$,<br>kernel='rbf',<br>probability=True	n_estimators= 100,<br>max_depth= 25	hidden_layer_sizes= $(1, 100)$,<br>activation='identity',<br>$\alpha = 0.0001$
PIN-4	$C = 100$,<br>$\gamma = 0.0001$,<br>kernel='linear',<br>probability=True	random_state=1,<br>max_depth= 15,<br>n_estimators= 500,<br>min_samples_split= 2,<br>min_samples_leaf= 1	hidden_layer_sizes= $(1, 100)$,<br>activation='logistic',<br>max_iter= 1000,<br>random_state= 1,<br>learning_rate='constant',<br>solver='lbfgs',<br>$\alpha = 0.001$

Table S2: AUC values for three ML algorithms: SVM, RF, and NN, and three sets of features, H&E (n=25), ERG (n=9), and PIN-4 (n=5). The features were selected by recursive feature elimination (RFE), see section 1.6 *Features sets of recursive feature elimination*. Only for PIN-4 stain, RFE significantly improved the AUC compared to Table 2. With five features selected for stain PIN-4, SVM and NN reached favorably high AUC values of 0.997 ± 0.009 and 0.992 ± 0.012, respectively. . The AUC scores are for ROC curves averaged over 100 Monte Carlo cross-validations with stratification at patient (n=48) level to avoid having cores coming from the same patient present in both training and validation set. Two algorithm, SVM and NN, yield mean AUCs which, within their standard deviations, are indistinguishable from a perfect score of 100%.

	**SVM**	**RF**	**NN**
H&E	0.83 ± 0.07	0.81 ± 0.07	0.82 ± 0.07
ERG	0.90 ± 0.05	0.87 ± 0.06	0.90 ± 0.05
PIN-4	$\mathbf{0.997 \pm 0.009}$	0.95 ± 0.04	$\mathbf{0.992 \pm 0.012}$

Table S3: Sensitivity (recall) averaged of 100 Monte Carlo random splits with stratification at patient (n=48) level. We applied three ML algorithms: SVM, RF, and NN, and three sets of features, H&E (n=25), ERG (n=9), and PIN-4 (n=5), as in Table S2. Note that, elevated expression of ERG has shown to occur only in a subset of approximately 50% of PCa cases [5, 6]. PIN-4 yields best sensitivity. Two algorithm, SVM and NN, yield mean sensitivities which, within their standard deviations, are indistinguishable from a perfect score of 100%.

	**SVM**	**RF**	**NN**
H&E	0.680 ± 0.142	0.538 ± 0.137	0.746 ± 0.136
ERG	0.521 ± 0.144	0.617 ± 0.155	0.670 ± 0.124
PIN-4	0.939 ± 0.081	0.825 ± 0.108	$\mathbf{0.961 \pm 0.068}$

Table S4: Number of malignant and benign cores stained with H&E, ERG, and PIN-4 and processed with the software QuPath. We punched 106 representative tissue cores, 38 malignant cores and 68 benign cores. Three stained cores had to be excluded from analysis due to poor quality.

**staining**	**recognized cores**	**malignant cores**	**benign cores**
H&E	105	38	67
ERG	105	37	68
PIN-4	105	38	67

Table S5: Abbreviation of predefined standard features in QuPath [1]. Five of the standard features are based on the intensity distribution and 13 are Haralick texture features.

**Intensity-based basic features (5)**	**Abbreviation**
Mean value	Mean
Standard deviation	Std
Minimum value	Min
Maximum value	Max
Median value	Median
**Intensity-based Haralick features (13)**	**Abbreviation**
Angular second moment	F0
Contrast	F1
Correlation	F2
Sum of squares	F3
Inverse difference moment	F4
Sum average	F5
Sum variance	F6
Sum entropy	F7
Entropy	F8
Difference variance	F9
Difference entropy	F10
Information measure of correlation 1	F11
Information measure of correlation 2	F12

Figures

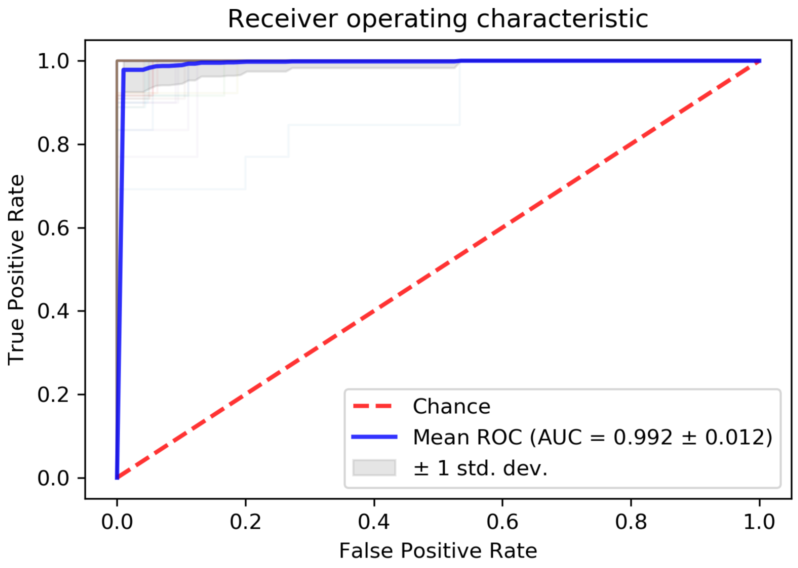

Figure S1: ROC curves of Monte Carlo cross-validation with 100 random splits. The blue curve denotes the mean ROC curve and the shaded gray area highlights its standard deviation. NN uses five features of the staining PIN-4 that are selected by recursive feature elimination (RFE), see section 1.6 *Features sets of recursive feature elimination*. The AUC 0.992 of the mean ROC curve (mean accuracy 98.2%) demonstrates the power of the features of stain PIN-4 to discriminate between malignant and benign tissue cores.

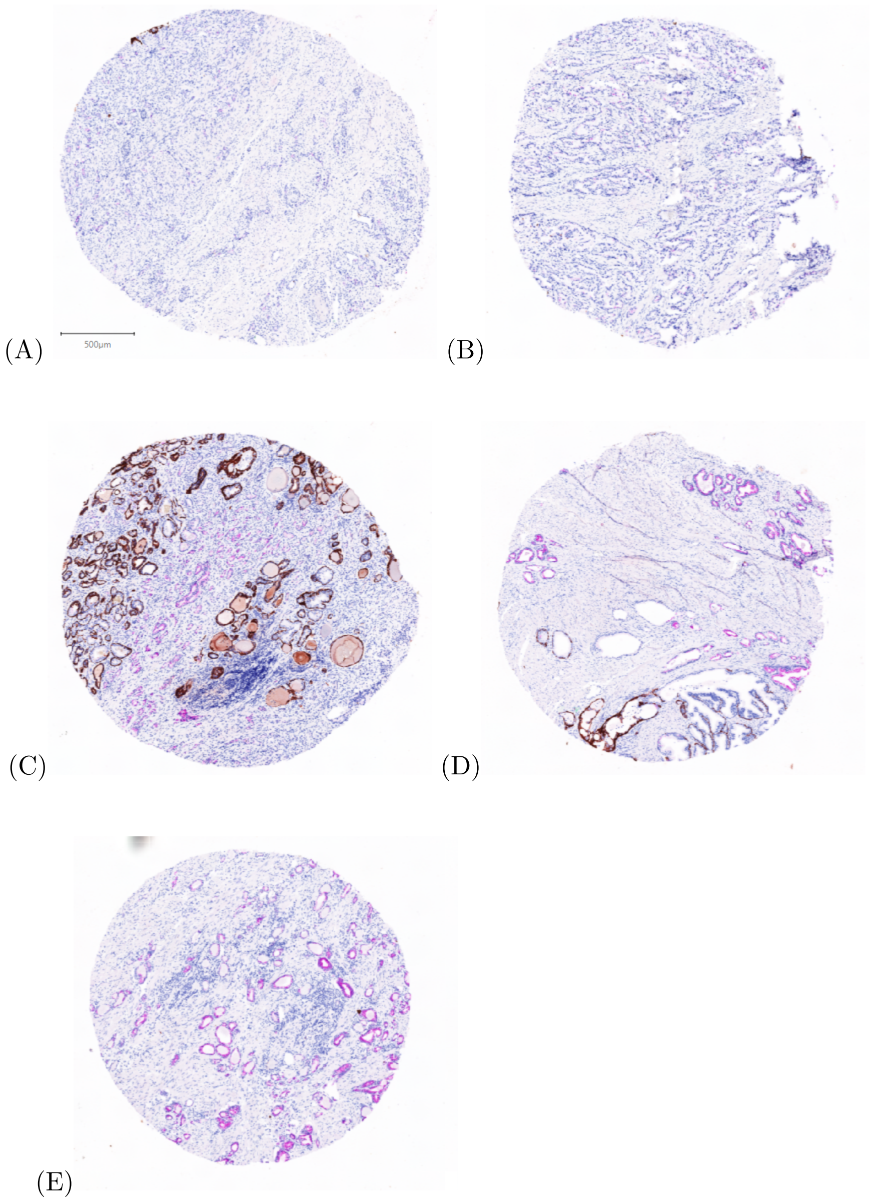

Figure S2: Malignant cores stained with PIN-4 and with Maximal Saturation below 0.953. Top left to bottom right, cores with ID/Maximum Saturation: (A) RPX1:1A/0.808, (B) RPX1:3C/0.889, (C) RPX1:5E/0.894, (D) RPX1:5B/0.919, and (E) RPX1:7A/0.922. The scalebar (500μm) in part A applies to all five cores. The cores represent high grade prostate cancer (two cores, A–B) and low grade prostate cancers (three cores, C–E) with heterogenous AMCR expression.

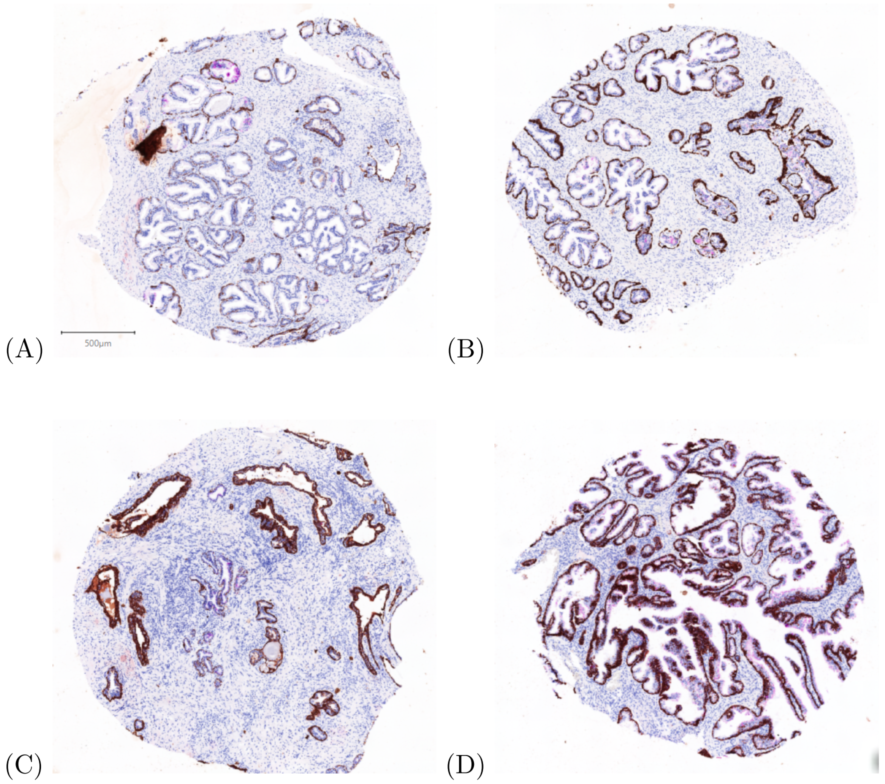

Figure S3: Benign cores stained with PIN-4 and with Maximum Saturation above 0.953. Top left to bottom right, cores with ID/Maximum Saturation: (A) RPX3:1C/0.979, (B) RPX3:2A/0.970, (C) RPX3:7A/0.967, and (D) RPX2:8C/0.964. The scalebar (500μm) in part A applies to all four cores. Intense dark brown stain leads to high values of Maximum Saturation. For RPX3:1C (core A), the staining artefact (dark spot in top left part of core A) leads to its high value of Maximum Saturation.

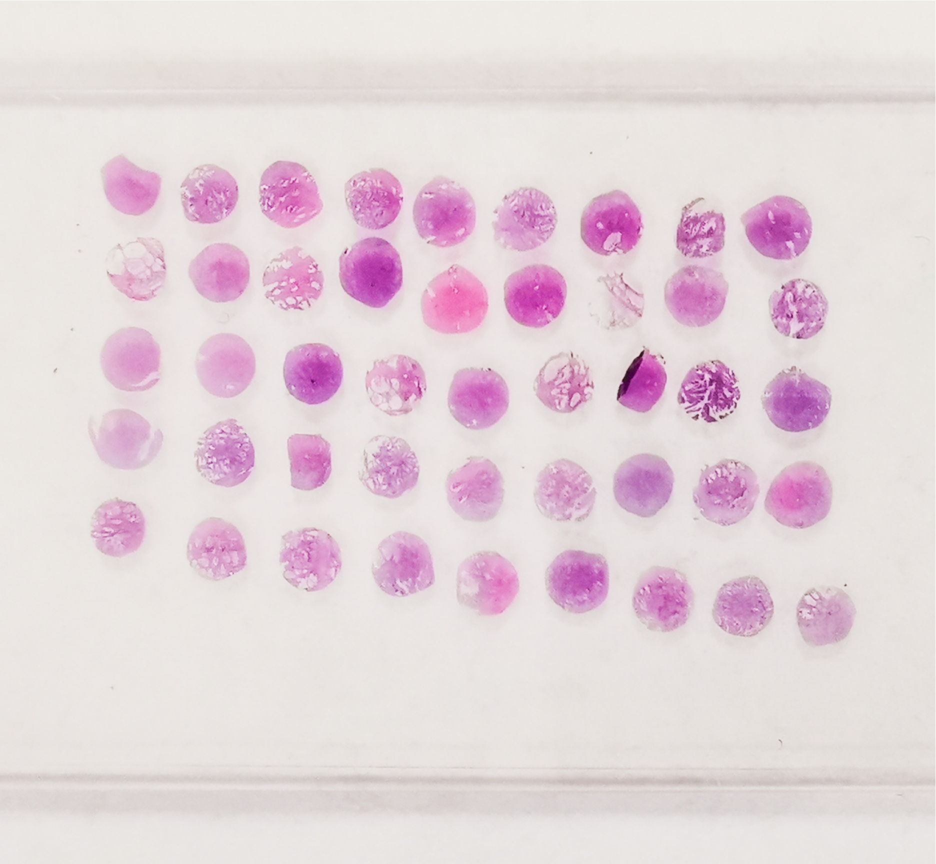

Figure S4: TMA slide, RPX2, established at the Institute of Pathology, University Hospital Frankfurt. The tissue block with 45 cores, each 2 mm in diameter, is stained with H&E. The array of cores has four rows, A-E, and nine columns, 1-9. Three cores, 1C, 1D, and 1C, from brain tissue are included for visual quality control.

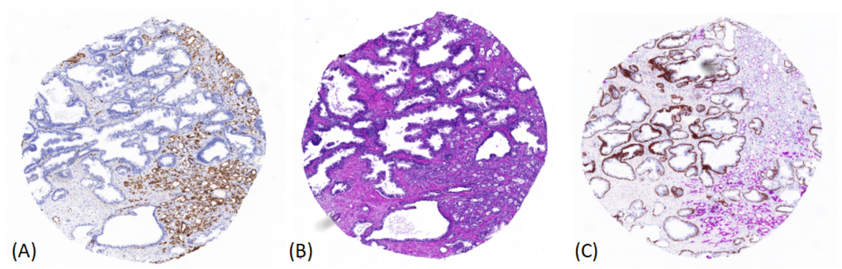

Figure S5: Exemplary cores with three stains: (A) ERG, (B) H&E , and (C) PIN-4.

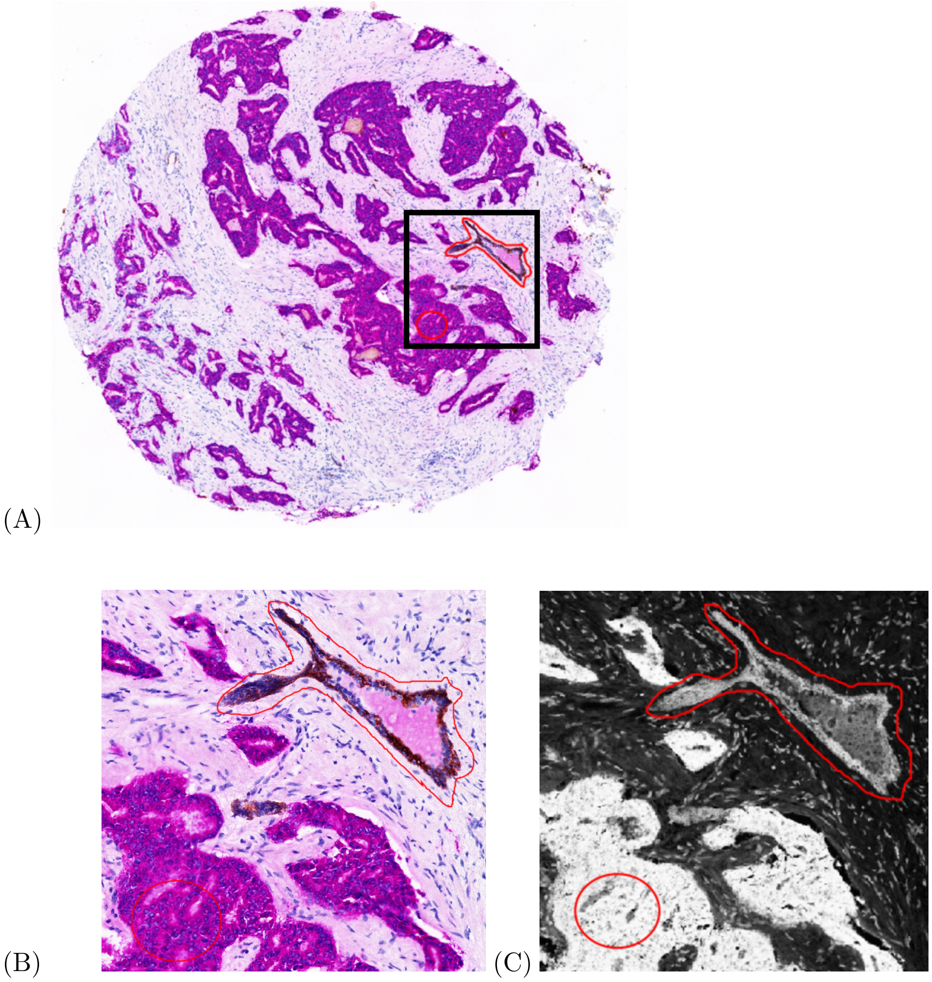

Figure S6: (A): Malignant core (ID: RPX1:7B) stained with PIN-4 and with the largest value of Maximum Saturation (1.0). A rectangle marks a zoom–in region. (B): Zoom into the marked region. In the upper right part, a red outline indicates position and shape of a gland. The gland is surrounded by brownish basal cells. The brownish basal cells appear darker than cells with a pure reddish cytoplasmic signal for AMACR; a red circle in the left bottom part indicates a region with dominant reddish signal. (C): Saturation of the marked region. For rgb-values, Red, Green, Blue, of a pixel the Saturation, s, is determined by $s = 1 - \min(Red, Green, Blue)/\max(Red, Green, Blue)$. The basal cells with brownish membranous signal (inside the outline of the gland) have a lower Saturation than cells with dominant reddish cytoplasmic signal for AMCAR (inside the circle, left bottom). The cells inside the outline of the gland vanished for the range of Saturation $0.91 \geq s \geq 1$, data not shown. The presence of a pixel with high Saturation indicates a pure reddish cytoplasmic signal for AMCAR without brownish signal for basal cells and may explain why Maximum Saturation was the best single indicator for malignancy, see text.

Ethics approval and consent to participate

Written informed consent was obtained from all patients and the study was approved by the institutional Review Boards of the UCT and the Ethical Committee at the University Hospital Frankfurt (project-number: SUG-4-2018).

The authors declare that they have no competing interests.

Author details

[1]Institute of Pathology, Ludwig-Maximilians University Munich, Thalkirchner Str. 36, 80337 Munich, Germany. [2]Institute of Pathology, Charité - Universitätsmedizin Berlin, corporate member of Freie Universität Berlin, Humboldt-Universität zu Berlin, 10117 Berlin, Germany. [3]Molecular Bioinformatics Group, Institute of Computer Science, Faculty of Computer Science and Mathematics, Robert-Mayer-Straße 11–15, 60325 Frankfurt am Main, Germany. [4]Dr. Senckenberg Institute for Pathology, Goethe University Frankfurt am Main, University Hospital Frankfurt, 60590 Frankfurt am Main, Germany. [5]Wildlab, University Hospital Frankfurt MVZ GmbH, 60590 Frankfurt am Main, Germany. [6]Frankfurt Institute for Advanced Studies (FIAS), Frankfurt Institute for Advanced Studies (FIAS), 60438 Frankfurt am Main, Germany. [7] Department of Urology, Goethe University Frankfurt am Main, University Hospital Frankfurt, 60590 Frankfurt am Main, Germany. [8]Department of Diagnostic and Interventional Radiology, Goethe University Frankfurt am Main, University Hospital Frankfurt, 60590 Frankfurt am Main, Germany. [9]Neurological Institute (Edinger Institute), University Hospital Frankfurt, 60590 Frankfurt am Main, Germany. [10]German Cancer Consortium (DKTK), German Cancer Research Center (DKFZ), 69120 Heidelberg, Germany. [11]University Cancer Center (UCT) Frankfurt, Frankfurt am Main, Germany. [12]Frankfurt Cancer Institute (FCI), University Hospital Frankfurt, 60590 Frankfurt am Main, Germany.

References

1. Bankhead, P., Loughrey, M.B., Fernández, J.A., Dombrowski, Y., McArt, D.G., Dunne, P.D., McQuaid, S., Gray, R.T., Murray, L.J., Coleman, H.G., James, J.A., Salto-Tellez, M., Hamilton, P.W.: QuPath: Open source software for digital pathology image analysis. Scientific Reports **7**(1), 1–7 (2017)
2. Bernatz, S., Ackermann, J., Mandel, P., Kaltenbach, B., Zhdanovich, Y., Harter, P.N., Döring, C., Hammerstingl, R., Bodelle, B., Smith, K., Bucher, A., Albrecht, M., Rosbach, N., Basten, L., Yel, I., Wenzel, M., Bankov, K., Koch, I., Chun, F.K.-H., Köllermann, J., Wild, P.J., Vogl, T.J.: Comparison of machine learning algorithms to predict clinically significant prostate cancer of the peripheral zone with multiparametric MRI using clinical assessment categories and radiomic features. European Radiology, 1–13 (2020)
3. Varma, M., Jasani, B.: Diagnostic utility of immunohistochemistry in morphologically difficult prostate cancer: review of current literature. Histopathology **47**(1), 1–16 (2005)
4. Bostwick, D.G., Qian, J.: High-grade prostatic intraepithelial neoplasia. Modern Pathology **17**(3), 360–379 (2004)
5. Scheble, V.J., Braun, M., Beroukhim, R., Mermel, C.H., Ruiz, C., Wilbertz, T., Stiedl, A.-C., Petersen, K., Reischl, M., Kuefer, R., Schilling, D., Fend, F., Kristiansen, G., Meyerson, M., Rubin, M.A., Bubendorf, L., Perner, S.: ERG rearrangement is specific to prostate cancer and does not occur in any other common tumor. Modern Pathology **23**(8), 1061–1067 (2010)
6. Perner, S., Rupp, N.J., Braun, M., Rubin, M.A., Moch, H., Dietel, M., Wernert, N., Jung, K., Stephan, C., Kristiansen, G.: Loss of SLC45A3 protein (prostein) expression in prostate cancer is associated with SLC45A3-ERG gene rearrangement and an unfavorable clinical course. International Journal of Cancer **132**(4), 807–812 (2013)
7. Ayala, G., Frolov, A., Chatterjee, D., He, D., Hilsenbeck, S., Ittmann, M.: Expression of ERG protein in prostate cancer: variability and biological correlates. Endocrine-Related Cancer **22**(3), 277 (2015)
8. Ruifrok, A.C., Johnston, D.A.: Quantification of histochemical staining by color deconvolution. Analytical and quantitative cytology and histology **23**(4), 291–299 (2001)
9. Guyon, I., Weston, J., Barnhill, S., Vapnik, V.: Gene selection for cancer classification using support vector machines. Machine learning **46**(1), 389–422 (2002)
10. Cheung, Y.K., Klotz, J.H.: The Mann Whitney Wilcoxon distribution using linked lists. Statistica Sinica, 805–813 (1997)
11. Mann, H.B., Whitney, D.R.: On a test of whether one of two random variables is stochastically larger than the other. The annals of mathematical statistics, 50–60 (1947)
12. Bamber, D.: The area above the ordinal dominance graph and the area below the receiver operating characteristic graph. Journal of mathematical psychology **12**(4), 387–415 (1975)
13. Mason, S.J., Graham, N.E.: Areas beneath the relative operating characteristics (roc) and relative operating levels (rol) curves: Statistical significance and interpretation. Quarterly Journal of the Royal Meteorological Society: A journal of the atmospheric sciences, applied meteorology and physical oceanography **128**(584), 2145–2166 (2002)
14. Hanley, J.A., McNeil, B.J.: The meaning and use of the area under a receiver operating characteristic (ROC) curve. Radiology **143**(1), 29–36 (1982)
15. Benjamini, Y., Yekutieli, D.: The control of the false discovery rate in multiple testing under dependency. Annals of Statistics, 1165–1188 (2001)
16. Pedregosa, F., Varoquaux, G., Gramfort, A., Michel, V., Thirion, B., Grisel, O., Blondel, M., Prettenhofer, P., Weiss, R., Dubourg, V., Vanderplas, J., Passos, A., Cournapeau, D., Brucher, M., Perrot, M., Duchesnay, E.: Scikit-learn: Machine learning in Python. The Journal of Machine Learning Research **12**, 2825–2830 (2011)
17. Van Rossum, G., Drake, F.L.: Python 3 Reference Manual. CreateSpace, Scotts Valley, CA (2009)
18. Kluyver, T., Ragan-Kelley, B., Pérez, F., Granger, Granger, B.E., Bussonnier, M., Frederic, J., Kelley, K., Hamrick, J.B., Grout, J., Corlay, S., Ivanov, P., Avila, D., Abdalla, S., Willing, C.e.a.: Jupyter Notebooks-a publishing format for reproducible computational workflows. In: Positioning and Power in Academic Publishing: Players, Agents and Agendas, Proceedings of the 20th Confernce on Electronic Publishing, pp. 87–90 (2016)

19. Virtanen, P., Gommers, R., Oliphant, T.E., Haberland, M., Reddy, T., Cournapeau, D., Burovski, E., Peterson, P., Weckesser, W., Bright, J.e.a.: SciPy 1.0: fundamental algorithms for scientific computing in Python. Nature Methods **17**(3), 261–272 (2020)
